# The Links Between Physical Activity, Metacognition, and Empathy Among Physiotherapy Students

**DOI:** 10.3390/healthcare13182350

**Published:** 2025-09-18

**Authors:** Anica Kuzmić, Manuela Filipec, Miro Jakovljević

**Affiliations:** 1 Department of Physiotherapy, University North, Jurja Križanića 31b, 42000 Varaždin, Croatia; akuzmic@unin.hr; 2 School of Medicine, University of Zagreb, 10000 Zagreb, Croatia; jakovljevic.miro@yahoo.com

**Keywords:** IPAQ-SF, Metacognitive Awareness Inventory, empathy quotient, students

## Abstract

**Background:** Physical activity, metacognitive awareness, and empathy are interconnected factors that play a significant role in the overall well-being of university students. Together, these elements contribute to the development of more self-aware, emotionally intelligent, and academically engaged students. The aim of this study is to explore the relationship between physical activity, empathy, and metacognition among physiotherapy students, as well as determining whether differences exist between undergraduate and graduate students. **Methods:** A cross-sectional study evaluated 468 physiotherapy students using the International Physical Activity Questionnaire—short version, the Metacognitive Awareness Inventory, and a shortened version of the Empathy Quotient supplemented with demographic questions. The respondents were students of undergraduate and graduate studies of physiotherapy, were male and female, and were between the ages of 18 and 25 years. **Results:** Higher levels of vigorous-intensity physical activity, walking, and total physical activity are significantly associated with increases in Declarative Knowledge (*p* = 0.000; *p* = 0.001; *p* = 0.000), Empathy Quotient (*p* = 0.029; *p* = 0.000; *p* = 0.006), and Cognitive Empathy (*p* = 0.002; *p* = 0.000; *p* = 0.001). Undergraduate students demonstrated higher levels of Declarative Knowledge (*p* = 0.000), whereas graduate students scored higher in Procedural Knowledge (*p* = 0.020), Planning (*p* = 0.000), Information Management Strategies (*p* = 0.000), and Evaluation (*p* = 0.005). Undergraduate students demonstrated higher overall empathy, cognitive empathy, and social skills (*p* = 0.000). **Conclusions:** This is the first study to examine this issue in the context of physiotherapy students. Our findings highlight the importance of creating integrated programs that promote physical activity, metacognitive awareness, and empathy concurrently among physiotherapy students. Enhancing metacognitive skills through targeted educational strategies helps students strengthen their critical thinking and self-regulation, enhance academic outcomes, and better prepare students for their professional role.

## 1. Introduction

Physical activity, though often considered primarily in relation to physical health, has been increasingly recognized for its significant role in promoting psychological well-being and cognitive development, aligning closely with the core tenets of positive psychology. In the context of positive psychology, physical activity serves as a powerful means of cultivating personal strengths, such as self-confidence, self-discipline, and emotional regulation [1]. These attributes support the development of positive self-perception and resilience, which are essential components related to flourishing and optimal human functioning. Engaging in regular physical activity strengthens students’ self-esteem, self-efficacy, and personal responsibility, while simultaneously enhancing cognitive functions such as memory, attention, and executive control—key components of academic performance [2,3]. From a positive psychology lens, metacognitive skills empower students to become autonomous, self-directed learners, enhancing their sense of competence and agency. These are foundational to a growth mindset and intrinsic motivation, both of which are associated with higher levels of engagement and satisfaction [2,4,5]. Students with strong metacognitive skills are better equipped to manage cognitive resources, recognize mistakes, and adjust their time and learning strategies—abilities that mirror the executive functions tied to both academic performance and psychological resilience [6,7,8]. Moreover, such awareness extends to emotional self-regulation, helping students manage stress, maintain focus, and stay goal-directed—outcomes that reinforce the broader aims of positive education [9]. Research suggests that higher levels of metacognitive awareness among students lead to more effective control over cognitive processes such as planning, managing acquired information, recognizing personal errors, and evaluating outcomes, all of which contribute to better academic performance [10]. It positively influences students’ problem-solving skills by increasing processing speed, decision-making abilities, attention, concentration, memory, and enhancing executive functions [11]. These traits fall under the domain of emotional intelligence, a core construct in positive psychology that contributes to effective communication, empathy-driven relationships, and prosocial behavior [12]. Empathy, in particular, enables students to understand and respond to others’ emotions, fostering compassion, mutual respect, and a supportive learning environment [13]. Empathy can be differentiated into cognitive empathy—the ability to understand others’ perspectives—and affective empathy—the capacity to emotionally resonate with others. Both dimensions are vital for constructive social interaction and community engagement, directly aligning with positive psychology’s emphasis on cultivating interpersonal strengths and prosocial behavior [12]. Students with high levels of empathy are more adept at conflict resolution, collaboration, and perspective-taking—all of which are linked to improved academic outcomes and long-term emotional well-being [12]. Physical activity acts as a catalyst for holistic development, enhancing cognitive, emotional, and social functioning in a way that resonates strongly with the goals of positive psychology [14,15,16]. Mandolesi et al., as well as Gooderham and Handy, point out that physical activity induces structural and functional changes in the brain, determining enormous benefit to both cognitive functioning and well-being [14,17]. Egli et al. found differences in exercise motivation between different-gendered college students. Males were motivated by intrinsic factors (strength, competition, and challenge) and females by extrinsic factors (i.e., weight management and appearance) [15]. Papageorgiou et al. emphasize that the ability to empathize with patients is a crucial component of effective healthcare [16]. Also, their results show that medical students’ empathy varies, with empathy being highest at the start of the medical course in the first year, declining to a low level in the third year and then rising again in the fourth and fifth years. There was a tendency for female students to have higher empathy scores compared to male students in each of the five years, with scores being significantly different in the second, third, and fourth years [16]. While Neumann et al. suggest that empathy declines during medical school, it compromises striving toward professionalism and may threaten healthcare quality [13]. Communicating with patients is an essential physiotherapy activity. It has a therapeutic effect and supports the patient’s healing process; it also has a positive effect on psychosocial outcomes (e.g., quality of life) and on objectively measurable outcome parameters [13]. Empathy also plays an important role in achieving patient-centeredness, comprising the “qualities of compassion, and responsiveness to the needs, values, and expressed preferences of the individual patient” [13]. Christov-Moore et al. suggest that there are differences in the capacity for empathy between males and females [12]. Females are portrayed as more nurturing and empathetic, while males are portrayed as less emotional and more cognitive [12]. Palmer et al. observed that in adult populations, metacognitive monitoring of memory and learning is affected by physical activity interventions, through both the enhancement and impairment of absolute and relative performance [18]. However, less well documented are the differences among physiotherapy students regarding gender. While there is evidence that empathy fluctuates during undergraduate medical training (students of medicine and dental medicine, as well as nursing students), there has been very little research looking at physiotherapy students’ empathy levels over their full course of study. Also, there are few data about the relationship between physical activity, empathy, and metacognition, which are essential for studying, clinical practice, and the overall well-being of physiotherapists. In contemporary education, where stress, burnout, and mental health concerns among students are rising, identifying accessible and holistic strategies that simultaneously enhance cognitive, emotional, and social resources is essential. Physical activity is a catalyst for fostering metacognition and empathy and it could be a sustainable intervention that benefits not only academic achievement but also future professional effectiveness and social well-being. By investigating the interconnected role of physical activity in promoting metacognitive skills, empathy, and academic success, this research could contribute to the development of evidence-based educational practices that align with the goals of positive psychology and address needs in both higher education and society at large. Although previous studies have demonstrated the positive effects of physical activity on cognition and well-being, as well as gender-related differences in exercise motivation, little is known about how these findings translate to physiotherapy students. Research on empathy has largely focused on medical and nursing students, while evidence concerning physiotherapy students remains scarce. Similarly, while physical activity has been linked to improvements in metacognitive processes, there is limited knowledge regarding its role in shaping metacognitive skills in physiotherapy students. Moreover, the interplay between physical activity, empathy, and metacognition—three factors that are essential for both academic success and professional effectiveness—has not been explored in this population. Addressing this gap is particularly relevant in light of rising levels of stress and well-being among students and may provide a basis for developing holistic, evidence-based educational strategies that enhance cognitive, emotional, and social resources in future physiotherapy professionals.

The aim of this study is to explore the relationship between physical activity, empathy, and metacognition among physiotherapy students, as well as to determine whether differences exist between undergraduate and graduate students.

## 2. Materials and Methods

### 2.1. Study Design and Participants

This is a cross-sectional study which used questionnaires to assess physical activity, metacognition and empathy in physiotherapy students. The study was conducted between March and November 2024. The study was conducted in accordance with the Declaration of Helsinki and was approved by the Ethics Committee of the University North (approval number: 2137-0336-07-24-1; date: 26 February 2024). Each participant provided written informed consent. In total, 468 participants were recruited from physiotherapy study courses in Croatia. The sample consisted of undergraduate and graduate students in physiotherapy, including males and females between the ages of 18 and 25 years. An announcement was posted electronically on the web page of each physiotherapy study course in Croatia, inviting students to participate in the study. There were 471 respondents, but three questionnaires were removed due to their partial completion.

### 2.2. Questionnaires

Participants were asked to indicate their age, gender, level of study, and year of study, as well as being asked to fill out three questionnaires about physical activity, metacognitive awareness, and empathy. The data were collected using valid and reliable questionnaires—The International Physical Activity Questionnaire—short version (IPAQ-SF) [19], the Metacognitive Awareness Inventory (MAI) [20], and a shortened version of the Empathy Quotient (EQ-28) [21]. In the literature, α reliabilities for the IPAQ-SF range from 0.60 to 0.647 [19,22,23], which is similar to our results (α = 0.616). Data relating to the Cronbach’s alfa of the nine subscales of MAI range from 0.75 to 0.91 in the literature (Procedural knowledge α = 0.77, Conditional knowledge α = 0.78, Planning α = 0.79, Information management strategies α = 0.77, Comprehension monitoring α = 0,80, Debugging strategies α = 0.79, Evaluation α = 0.75, and Overall metacognitive awareness α = 0.91) [20,24]. Values of the α reliabilities of the nine subscales of MAI are similar in our sample and range from 0.73 to 0.88. (Procedural knowledge α = 0.74, Conditional knowledge α = 0.76, Planning α = 0.73, Information management strategies α = 0.75, Comprehension monitoring α = 0.81, Debugging strategies α = 0.76, Evaluation α = 0.73, and Overall metacognitive awareness α = 0.88). Also, data relating to the Cronbach alfa of the four subscales of EQ-28 range from 0.570 to 0.850 (Cognitive Empathy α = 0.840, Emotional Reactivity α = 0.760, Social Skills α = 0.570, and Overall empathy quotient α = 0.850) [21,25], which is similar to our results of the α reliabilities of the four subscales of EQ-28, which range from 0.535 to 0.899 (Cognitive Empathy α = 0.899, Emotional Reactivity α = 0.752, Social Skills α = 0.535, and Overall empathy quotient α = 0.872).

The IPAQ-SF is a 7-item self-reported measure of physical activity comprising four categories—walking, moderate-intensity activities, vigorous-intensity activities, and total physical activity. There were three levels of physical activity to classify—low, moderate, and high. All continuous scores are expressed in MET-minutes/week and are scored according to the Guidelines for Data Processing and Analysis of the International Physical Activity Questionnaire [26]. Data collected with IPAQ were computed by weighting each type of activity by its energy requirements defined in METs to yield a score in MET–minutes [26]. METs are multiples of the resting metabolic rate, and a MET-minute is computed by multiplying the MET score of an activity by the minutes performed [26]. The data were expressed in minutes/day and days/week. IPAQ-SF responses were converted to MET minutes per week (MET-min/wk) according to the IPAQ scoring protocol [26]; total minutes over the last seven days spent on vigorous activity, moderate-intensity activity, and walking were multiplied by 3.3, 4, and 8, respectively, in order to create MET scores for each activity level [26]. MET scores across the three sub-components were summed to indicate overall physical activity, as follows: walking MET-minutes/week = 3.3 × walking minutes × walking days; moderate MET-minutes/week = 4.0 × moderate-intensity activity minutes × moderate days; vigorous MET-minutes/week = 8.0 × vigorous-intensity activity minutes × vigorous-intensity days; total physical activity MET-minutes/week = sum of walking + moderate + vigorous MET minutes/week scores [26].

The Metacognitive Awareness Inventory (MAI) is a self-assessment tool used to measure students’ metacognitive abilities. It consists of 52 items and nine subscales (Declarative knowledge, Procedural knowledge, Conditional knowledge, Planning, Information management strategies, Comprehension monitoring, Debugging strategies, Evaluation, and Overall metacognitive awareness). Participants respond with “true” if they believe the statement reflects their personal experience, or with “false” if they believe it does not. A “true” response is scored as “1”, while a “false” response is scored as “0”. The total score for each category is then calculated. Higher scores indicate greater metacognitive awareness for subscales and overall metacognition [20].

The shortened version of the Empathy Quotient (EQ-28) is a self-report measure of empathy. It consists of 28 items and four subscales (Cognitive Empathy, Emotional Reactivity, Social Skills, and Overall empathy quotient). Participants were asked to choose how strongly they agree or disagree with each statement. The answers were offered on a 4-point scale from strongly agree to slightly agree to slightly disagree to strongly disagree. The items are scored 0, 1, or 2, with participants receiving 0 for a non-empathic response, or marks of 1 or 2 depending on the strength of an empathic response. Higher scores indicate higher levels of empathy [21].

### 2.3. Data Analysis

The Statistical Package IBM Corp. (released in 2019) IBM SPSS Statistics for Windows, Version 26.0. Armonk, NY, USA. was used to conduct data analysis.

A power analysis was conducted to determine the required sample size for the study. The sample size was calculated using a two-tailed correlation analysis with an estimated effect size of r = 0.05, a significance level of α = 0.05, and a power of 80%. The power analysis indicated that 456 participants were required.

The Kolmogorov–Smirnov and Shapiro–Wilk tests were used to test the normality of the distribution. Furthermore, the research was conducted in two research groups (undergraduate and graduate students) and subgroups according to gender and year of study. To test the differences between groups (undergraduate and graduate students) and subgroups (female and male students), as well as between the IPAQ-SF, MAI, and EQ-28 variables, the Chi-square test and the Mann–Whitney U rank sum test were used. While testing differences between groups (undergraduate and graduate students) and year of study, the Kruskal–Wallis test was used. The correlations between the observed questionnaire components were tested using Spearman’s correlation coefficient. All results were interpreted at the level of statistical significance of *p* < 0.05.

## 3. Results

Table 1 presents the demographic data of the participants.

The results show that higher levels of vigorous-intensity physical activity, walking, and total physical activity are significantly associated with increases in Declarative knowledge (*p* = 0.000; *p* = 0.001; *p* = 0.000), Empathy quotient (*p* = 0.029; *p* = 0.000; *p* = 0.006), and Cognitive empathy (*p* = 0.002; *p* = 0.000; *p* = 0.001). In contrast, moderate-intensity physical activity did not show a significant effect on the scores of MAI items or EQ-28 items (Table 2).

Furthermore, the results suggest a positive correlation between vigorous-intensity physical activity, walking, and total physical activity and Declarative knowledge (r = 0.247; r = 0.170; r = 0.200), Empathy quotient (r = 0.122; r = 0.183; r = 0.145), and Cognitive empathy (r = 0.166; r = 0.213; r = 0.168), indicating that higher engagement in these forms of physical activity may be associated with enhanced cognitive and empathic capacities (Table 2).

Statistically significant differences were found in the intensity of physical activity in relation to the level of study and gender. There are differences in vigorous-intensity physical activity, walking, and total physical activity in relation to the year of study, indicating that undergraduate students have a high and moderate level of physical activity (*p* = 0.000) compared to graduate students (Table 3). Furthermore, male participants had a higher total physical activity (*p* = 0.028) compared to female participants.

Statistically significant differences were observed in Declarative knowledge (*p* = 0.000), Procedural knowledge (*p* = 0.020), Planning (*p* = 0.000), Information management strategies (*p* = 0.000), and Evaluation (*p* = 0.005) in regard to the level of study. Undergraduate students demonstrated higher levels of Declarative knowledge, whereas graduate students scored higher with Procedural knowledge, Planning, Information management strategies, and Evaluation. Furthermore, a statistically significant difference was found in relation to Information management strategies (*p* = 0.015), with female students exhibiting more developed strategies in this domain (Table 4).

Significant differences were observed in the Overall empathy quotient, Cognitive Empathy, and Social Skills (*p* = 0.000) with regard to the level of study. Undergraduate students demonstrated higher Overall empathy, Cognitive empathy, and Social skills compared to graduate students. Additionally, significant differences were found in the Empathy quotient (*p* = 0.000) and Emotional reactivity (*p*= 0.000), with female students exhibiting higher levels of Overall empathy and Emotional reactivity (Table 5).

A statistically significant difference in academic success was observed between study levels, with graduate students achieving a higher average grade at the end of the year compared to undergraduate students (*p* = 0.000) (Table 6).

## 4. Discussion

The main findings in our study are that higher levels of vigorous-intensity physical activity, walking, and total physical activity are significantly associated with increases in Declarative knowledge, Empathy quotient, and Cognitive empathy; undergraduate students demonstrated higher levels of Declarative knowledge, whereas graduate students scored higher in Procedural knowledge, Planning, Information management strategies, and Evaluation; and undergraduate students demonstrated higher Overall empathy, Cognitive empathy, and Social skills. 

Positive psychology, as a science of human flourishing, emphasizes the cultivation of strengths, optimal functioning, and well-being across cognitive, emotional, and social domains. In this context, physical activity (PA) is not merely a means to maintain physical fitness but a powerful tool for enhancing psychological and academic functioning, particularly among university students [17]. Furthermore, structural changes following PA have been related to academic achievement in comparison to sedentary individuals [17], supporting the “Engagement” and “Achievement” dimensions of the PERMA model in positive psychology. Increased physical activity may contribute to better cognitive empathy, which involves understanding another person’s thoughts and feelings [27] and metacognitive awareness [14]. Both constructs are closely aligned with emotional intelligence and self-regulated learning, which are central to the development of resilience, compassion, and adaptive functioning, which are key objectives of positive psychology in education [17].

Our results show that higher levels of physical activity, particularly vigorous-intensity activities and walking, appear to be positively associated with cognitive and emotional outcomes. Specifically, individuals engaging in more frequent or intense physical activity tend to exhibit greater Declarative knowledge, suggesting enhanced cognitive functioning or improved learning capacity. Additionally, increased physical activity is linked to higher Empathy quotient scores and greater Cognitive empathy, indicating a potential role of physical engagement in fostering social understanding and emotional intelligence. These findings highlight the multifaceted benefits of physical activity, extending beyond physical health to include cognitive and psychosocial domains. Also, it emphasizes the cultivation of human strengths, well-being, and optimal functioning. Physical activity may serve as a practical means to enhance key dimensions of flourishing—such as engagement, positive relationships, and personal growth—thus reinforcing the notion that movement and well-being are deeply interconnected across cognitive, emotional, and social domains. These multidimensional benefits reflect the core goals of positive psychology—promoting not only competence but also meaning, connection, and well-being in individuals’ lives.

Furthermore, in our study, male university students demonstrated higher levels of more vigorous physical activity compared to female students, which is consistent with research by Keating et al. and Huang et al. [28,29]. Also, Huang et al. indicated that male college students participated in more vigorous activities than female students [29]. This disparity may be influenced by a combination of biological, psychological, social, and environmental factors, which interact throughout development and across educational settings. Self-efficacy also plays a significant role, with males generally reporting higher confidence levels in their physical competence and sports performance, which positively influences their participation [28]. Additionally, among graduate students, an increase in participation in low-intensity physical activity and a decrease in engagement in higher-intensity physical activities were observed, which is consistent with the findings reported by Egli [15] and Buckworth [30]. While physical activity is widely recognized as a crucial factor in promoting physical health, cognitive function, mental well-being, and academic performance, participation levels among students remain suboptimal. Huang et al. found that college students reported that they became less physically active over time [29]. Keating et al. [28] found that 40% to 50% of students are not physically active, while Bajuaifer and Alrashdi [31] point out that physical activity levels among university students are concerningly low, with many failing to meet the WHO-recommended PA cutoffs [31].

Safi et al. [32] and Bismarck Ndupu et al. [33] identify a lack of management support in educational institutions, academic obligations, social support, and time and financial constraints as significant barriers to greater student participation in physical activity. The results highlight the need for targeted interventions and physical activity promotion programs, especially among senior students, to encourage participation in physical activity and improve the physical and mental health of the student population. This is essential for developing more equitable and effective health promotion strategies in university settings. Positive psychology emphasizes the importance of supportive environments that nurture autonomy, purpose, and intrinsic motivation [17]. Hence, university settings should foster institutional cultures that prioritize well-being through accessible, inclusive, and engaging physical activity programs for students.

The results of our study show that undergraduate physiotherapy students have more developed Declarative knowledge, whereas graduate students achieve higher results in Procedural knowledge, Planning, Strategies, Information management, and Evaluation. Declarative knowledge, i.e., knowledge relating to facts and concepts (“knowing what”), tends to develop more frequently in the earlier stages of education, while as students advance, especially at the graduate level, the focus increasingly shifts towards Procedural knowledge (“knowing how”), the use of learning strategies, understanding when and why to use those strategies (conditional knowledge), planning, and evaluation [34]. Declarative knowledge forms the foundation for understanding and applying Procedural knowledge, while Procedural knowledge is crucial for the development of practical skills [34]. Research emphasizes the importance of the balanced development of both types of knowledge in higher education, with Procedural knowledge being especially encouraged through research activities and projects, which are typical of graduate studies [35]. Furthermore, metacognitive awareness promotes reflection on one’s own thoughts, which is key to the development and analysis of critical thinking in physiotherapy students. Understanding one’s cognitive processes enables students to approach problem-solving in a structured way, apply different strategies, and adapt their approach to find optimal solutions. Students who actively engage in reflection on their learning strategies can identify the most effective methods and adjust them to their individual needs [36]. Metacognitive skills are especially important in physiotherapy, ranging from better learning abilities to becoming a better clinician. Metacognitive awareness in physiotherapy students leads to improved critical thinking, ensuring self-awareness and the use of self-regulated learning strategies to achieve a better cognitive performance in clinical settings [37]. It was also observed that female students have more developed Information management strategies. This finding is consistent with the study by Lopez Vincent, which showed that female students more frequently use a variety of information sources, more effectively evaluate and select information, and are more inclined to engage in complex forms of information processing and organization compared to male students [38]. Supporting individual learning styles and strengths, as promoted by positive psychology, can create more inclusive and effective educational practices that maximize each student’s potential. To achieve balance, it is recommended to integrate practical experiences, an interdisciplinary approach, real-life examples, and opportunities for independent learning. Such an approach enables students to gain a comprehensive understanding and develop the practical competencies necessary for professional success [6].

The results of our study indicate differences in empathy among physiotherapy students based on their level of study and gender. It was found that undergraduate students demonstrate higher Cognitive empathy, Social skills, and Empathy quotient compared to graduate students. These findings are consistent with previous research suggesting that empathy may decline in the later years of study, often attributed to academic workload, increased stress, or the professionalization of the approach to patients [13,16,39]. On the one hand, students at the beginning of their education, i.e., during undergraduate studies, still tend to possess strong intrinsic motivation to help and a stronger identification with the role of a future healthcare professional. On the other hand, as they progress through their studies and face greater clinical challenges, their empathy may decline due to emotional exhaustion or the development of emotional distancing mechanisms [40]. These results point to the need for targeted empathy-preservation strategies, such as reflective practices, narrative medicine, and emotional resilience training—interventions grounded in positive psychology’s emphasis on compassion and emotional flourishing. Also, positive psychology offers valuable insights here; by reinforcing purpose, compassion, and relational connectedness, it is possible to maintain empathy even amid professionalization pressures [41]. Additionally, the results show a statistically significant difference in the Empathy quotient and Emotional reactivity based on gender, with female students scoring higher in both categories. These findings are consistent with previous studies indicating that women generally demonstrate higher levels of empathy compared to men, which may be linked to biological, social, and cultural factors [12,42]. Women are often socialized to be more attuned to the emotional needs of others, which can be reflected in their professional competencies and approach to patient care. These findings suggest that women are more sensitive to emotionally salient stimuli, and this sensitivity extends beyond early emotional reactivity to later emotion regulation processes [43]. Gender differences in empathy are also reflected in patterns of brain activity. Women generally show greater activation in affective and sensory regions of the brain during empathy-related tasks, suggesting a more emotionally resonant empathic response. Men, on the other hand, tend to recruit brain regions associated with cognitive processing and contextual evaluation. These differences point to distinct but complementary neural strategies for empathy in men and women [44]. It is important to emphasize that preserving and developing empathy throughout the education of healthcare professionals has direct implications for the quality of care and patient satisfaction. Therefore, future interventions and changes in the educational system should aim to strengthen and maintain empathy in physiotherapy students, especially during graduate studies, to prevent its decline and ensure a high level of professional competence.

Our results show that graduate physiotherapy students achieve a higher average grade at the end of the year (academic success) compared to undergraduate students. This is consistent with studies by Callo et al. and Al Shawwa showing that advanced learners benefit from greater autonomy, experience, and metacognitive maturity [41,45]. Also, this aligns with positive psychology’s view of academic success not merely as performance, but as a reflection of personal growth, mastery, and self-determined engagement [41]. Graduate studies require more advanced competencies, a higher level of independence, and greater experience, which may contribute to a better academic performance compared to undergraduate studies, which are more focused on acquiring fundamental knowledge and skills [46]. Pintrich suggests that self-regulation and time management are strongly associated with success in graduate programs [6]. Students who enroll in graduate studies often have more developed learning strategies and better time management skills due to their previous experience, which results in better academic success [47]. Moreover, graduate students tend to use more advanced learning strategies, where metacognitive abilities, resource management strategies, and support from institutions and society are strong predictors of academic success [48]. These factors collectively enable students to be proactive, persistent, and efficient in their academic development. Such results are most often explained by accumulated knowledge, higher motivation, and more developed academic and professional skills among physiotherapy students at higher levels of education [48].

Our findings emphasize the need for integrated educational strategies that simultaneously support physical activity, metacognitive development, and empathy cultivation among physiotherapy students. Integrating structured exercise programs into the curriculum could serve as a cost-effective and accessible strategy to enhance both cognitive and socio-emotional competencies. For example, universities could offer regular group fitness sessions, workshops on mindful movement, or incentivized physical activity challenges tailored to physiotherapy students’ schedules. These interventions can be adapted to meet the developmental needs of both undergraduate and graduate students, with undergraduates benefiting from foundational skills in self-regulation, collaborative learning, and reflective thinking, while graduate students focus on advanced applications such as clinical reasoning, professional decision-making, and complex patient-centered care. Understanding the role of empathy in student development can help educators design targeted interventions that foster both cognitive and socio-emotional growth. For example, incorporating structured, goal-oriented, or group-based activities can enhance intrinsic motivation, collaborative engagement, and empathetic skills, supporting both academic performance and future clinical practice. Students who develop stronger self-monitoring skills and empathic understanding are likely to communicate more effectively with patients, make more reflective clinical decisions, and adopt patient-centered care practices. Integrating reflective exercises, peer feedback, or case-based learning alongside physical activity interventions may further consolidate these competencies, supporting skill development in both undergraduate and graduate students. Structured physical activity can act as a preventive strategy, improving well-being while simultaneously enhancing academic and clinical performance. By embedding these findings into evidence-based educational practices, physiotherapy programs can support students in achieving holistic development, also equipping them with the cognitive, emotional, and interpersonal capacities required for professional success.

Programs that embed practical experience, interdisciplinary learning, self-reflection, and real-world application can enhance students’ critical thinking, self-regulation, and interpersonal competence—core elements of both academic and professional excellence. Ultimately, such a holistic approach aligns with the principles of positive education, which seeks to promote well-being and learning concurrently, empowering students not only to succeed academically but to flourish as resilient, empathetic, and capable health professionals [41].

Future research should continue to explore the impact of integrated, strength-based interventions on students’ academic success, clinical competence, and long-term well-being. This would contribute to the advancement of educational policies that reflect the core values of positive psychology—fostering environments where students thrive cognitively, emotionally, and socially.

The limitations of the study refer to the inability to compare results with other biomedical (e.g., medicine, nursing, and occupational therapy) programs in order to gain insights into the similarities and differences in patterns of physical activity, metacognition, and empathy across various healthcare professions. Also, a limitation of this cross-sectional design is that it does not allow for the establishment of causal relationships. Consequently, while the study identifies links between physical activity, metacognition, and empathy, it cannot determine the directionality of these effects. Additionally, differences in gender-related behaviors or academic stage might affect physical activity patterns, cognitive strategies, and empathic abilities. Future studies should therefore adopt longitudinal or experimental designs and control for these variables to provide more comprehensive conclusions.

## 5. Conclusions

This study provides important insights into the role of physical activity in fostering both cognitive and socio-emotional competencies among physiotherapy students. By demonstrating significant associations between vigorous physical activity, walking, and total physical activity and higher levels of declarative knowledge, empathy, and cognitive empathy, the research highlights the multifaceted benefits of active lifestyles in academic contexts. Furthermore, the differences observed between undergraduate and graduate students underscore the relevance of the educational stage in the development of metacognitive and empathic skills, pointing to the designing of more effective educational strategies and student support systems. 

The value of this study lies in its contribution to the growing evidence that physical activity functions not only as a health-promoting behavior but also as a facilitator of core psychological capacities that underlie academic success and professional competence. These findings support the integration of structured physical activity opportunities, metacognitive training, and empathy development into physiotherapy curricula, fostering students’ holistic growth. Such an approach not only enhances academic performance but also equips future professionals with the cognitive flexibility, emotional intelligence, and interpersonal sensitivity essential for effective clinical practice. Ultimately, the results of this research may help in the development of recommendations and guidelines for educators and policymakers seeking to design learning environments that align physical, cognitive, and emotional development, with clear benefits for both students and the broader healthcare system.

## Figures and Tables

**Table 1:** Demographic data of the participants.

		N (%)
Gender	Male	127 (27.1)
	Female	341 (72.9)
	All	468 (100)
Employment	Yes	138 (29.5)
	No	330 (70.5)
BMI	Underweight	27 (5.8)
	Normal weight	343 (73.3)
	Pre-obesity	32 (6.8)
	Obesity class I	54 (11.5)
	Obesity class II	12 (2.6)
Level of study	Year of study	
Undergraduate	1st	78 (16.7)
	2nd	171 (36.5)
	3rd	132 (28.2)
Graduate	1st	43 (9.2)
	2nd	44 (9.4)

**Table 2:** Relationship between physical activity, MAI items, and EQ-28 items.

IPAQ Items	Vigorous-Intensity Physical Activity			Moderate-Intensity Physical Activity			Walking			Total Physical Activity		
	Low Level	Moderate Level	High Level	Low Level	Moderate Level	High Level	Low Level	Moderate Level	High Level	Low Level	Moderate Level	High Level
**MAI items**	r *; *p* **											
Declarative knowledge	0.247; 0.000			0.078; 0.090			0.170; 0.001			0.200; 0.000		
Procedural knowledge	0.007; 0.568			0.079; 0.109			0.041; 0.112			−0.015; 0.295		
Conditional knowledge	0.051; 0.328			0.054; 0.430			0.085; 0.116			0.065: 0.214		
Planning	0.027, 0.066			0.036; 0.403			−0.027; 0.429			−0.013; 0.084		
Information management strategies	−0.049; 0.524			−0.001; 1.000			0.015; 0.835			−0.026; 0.097		
Comprehension monitoring	0.071; 0.302			0.070; 0.270			0.025; 0.340			0.039; 0.518		
Debugging strategies	−0.065; 0.264			−0.021; 0.309			−0.003; 0.767			−0.056; 0.289		
Evaluation	0.054; 0.459			0.078; 0.233			0.057; 0.205			0.032; 0.769		
Overall metacognitive awareness	0.093 *; 0.125			0.068; 0.333			0.078; 0.129			0.070; 0.094		
**EQ-28 items**	r *; *p* **											
Empathy quotient	0.114; 0.029			0.079; 0.145			0.183; 0.000			0.145; 0.006		
Cognitive Empathy	0.166; 0.002			0.107; 0.063			0.213; 0.000			0.168; 0.001		
Emotional Reactivity	−0.020; 0.804			0.016; 0.417			0.068; 0.095			0.055; 0.499		
Social Skills	0.092; 0.074			0.053; 0.430			0.090; 0.130			0.081; 0.096		

* Spearman’s correlation coefficient; ** Kruskal–Wallis test.

**Table 3:** IPAQ by level of study and gender.

IPAQ Items		All	Level of Study		*p* *	Male	Female	*p* *
			Undergraduate	Graduate		N (%)	N (%)	
		N (%)	N (%)	N (%)				
Vigorous-intensity physical activity	Low level	155 (33.1)	97 (25.5)	58 (66.7)	0.000	28 (22.0%)	127 (37.2%)	0.000
	Moderate level	172 (36.8)	159 (41.7)	13 (14.9)		42 (33.1%)	130 (38.1%)	
	High level	141 (30.1)	125 (32.8)	16 (18.4)		57 (44.9%)	84 (24.6%)	
Moderate-intensity physical activity	Low level	225 (48.1)	180 (47.2)	45 (51.7)	0.406	54 (42.5%)	171 (50.1%)	0.321
	Moderate level	200 (42.7)	168 (44.1)	32 (36.8)		59 (46.5%)	141 (41.3%)	
	High level	43 (9.2)	33 (8.7)	10 (11.5)		14 (11.0%)	29 (8.5%)	
Walking	Low level	168 (35.9)	114 (29.9)	54 (62.1)	0.000	43 (33.9%)	125 (36.7%)	0.799
	Moderate level	261 (55.8)	236 (61.9)	25 (28.7)		74 (58.3%)	187 (54.8%)	
	High level	39 (8.3)	31 (8.1)	8 (9.2)		10 (7.9%)	29 (8.5%)	
Total physical activity	Low level	23 (4.9)	9 (2.4)	14 (16.1)	0.000	6 (4.7%)	17 (5.0%)	0.028
	Moderate level	154 (32.9)	115 (30.2)	39 (44.8)		30 (23.6%)	124 (36.4%)	
	High level	291 (62.2)	257 (67.5)	34 (39.1)		91 (71.7%)	200 (58.7%)	

* Chi-square test.

**Table 4:** MAI results by level of study and gender.

MAI Items	All	Undergraduate Level	Graduate Level		Male	Female	
	Median (IQR)			*p* *	Median (IQR)		*p* *
Declarative knowledge	7.00 (5.00–8.00)	7.00 (5.00–8.00)	6.00 (3.00–8.00)	0.000	7.00 (5.00–8.00)	7.00 (5.00–8.00)	0.988
Procedural knowledge	3.00 (3.00–4.00)	3.00 (2.00–4.00)	4.00 (3.00–4.00)	0.020	3.00 (2.00–4.00)	3.00 (3.00–4.00)	0.966
Conditional knowledge	4.00 (3.00–5.00)	4.00 (3.00–5.00)	4.00 (3.00–5.00)	0.255	4.00 (3.00–5.00)	4.00 (3.00–5.00)	0.752
Planning	6.00 (4.00–7.00)	5.00 (4.00–6.00)	7.00 (5.00–7.00)	0.000	6.00 (4.00–7.00)	6.00 (4.00–7.00)	0.910
Information management strategies	9.00 (7.00–10.00)	9.00 (7.00–9.00)	9.00 (9.00–10.00)	0.000	8.00 (7.00–9.00)	9.00 (8.00–10.00)	0.015
Comprehension monitoring	6.00 (5.00–7.00)	6.00 (5.00–7.00)	6.00 (5.00–7.00)	0.118	6.00 (5.00–7.00)	6.00 (5.00–7.00)	0.326
Debugging strategies	5.00 (4.00–5.00)	5.00 (4.00–5.00)	5.00 (4.00–5.00)	0.245	5.00 (4.00–5.00)	5.00 (4.00–5.00)	0.230
Evaluation	5.00 (4.00–6.00)	5.00 (4.00–6.00)	5.00 (4.00–6.00)	0.005	5.00 (4.00–6.00)	5.00 (4.00–6.00)	0.905
Overall metacognitive awareness	42.50 (37.00–48.00)	42.00 (36.00–48.00)	44.00 (38.00–49.00)	0.058	41.00 (36.00–48.00)	43.00 (37.00–48.00)	0.488

* Mann–Whitney U.

**Table 5:** EQ-28 by level of study and gender.

EQ-28 Items	Undergraduate Level	Graduate Level	*p* *	Male	Female	*p* *
	Median (IQR)					
Empathy quotient	31.00 (24.00–37.00)	24.00 (20.00–33.00)	0.000	28.00 (21.00–33.00)	31.00 (24.00–38.00)	0.000
Cognitive empathy	12.00 (9.00–16.00)	10.00 (5.00–14.00)	0.000	11.00 (7.00–16.00)	12.00 (9.00–15.00)	0.229
Emotional reactivity	13.00 (10.00–16.00)	12.00 (10.00–15.00)	0.277	11.00 (7.00–13.00)	14.00 (11.00–16.00)	0.000
Social skills	5.00 (4.00–7.00)	4.00 (3.00–6.00)	0.000	5.00 (3.00–7.00)	5.00 (3.00–7.00)	0.917

* Mann–Whitney U.

**Table 6:** Academic success.

	Undergraduate Level			Graduate Level	
	1st Year (N = 78)	2nd Year (N = 171)	3rd Year (N = 132)	1st Year (N = 43)	2nd Year (N = 44)
	Median (IQR)				
Average grade at the end of the year	4.00 (3.80–4.37)	3.90 (3.58–4.00)	4.00 (3.55–4.10)	4.40 (4.00–4.70)	4.30 (3.90–4.50)
*p* *	0.000				

* Kruskal–Wallis Test.

## Data Availability

The data presented in this study are available on request from the corresponding author. The data are not publicly available due to privacy or ethical restrictions.
